# Developmental and Reproductive Effects of Iron Oxide Nanoparticles in *Arabidopsis thaliana*

**DOI:** 10.3390/ijms161024174

**Published:** 2015-10-13

**Authors:** Sergey Bombin, Mitchell LeFebvre, Jennifer Sherwood, Yaolin Xu, Yuping Bao, Katrina M. Ramonell

**Affiliations:** 1 Department of Biological Sciences, University of Alabama, Box 870344, Tuscaloosa, AL 35487, USA; E-Mails: sbombin@crimson.ua.edu (S.B.); mnlefebvre@crimson.ua.edu (M.L.); 2 Department of Chemical and Biological Engineering, University of Alabama, Box 870203, Tuscaloosa, AL 35487, USA; E-Mails: jasherwood@crimson.us.edu (J.S.); yxu22@crimson.us.edu (Y.X.)

**Keywords:** nanoparticle, iron oxide, *Arabidopsis thaliana*, phytotoxicity, pollen viability, plant development, seed yield

## Abstract

Increasing use of iron oxide nanoparticles in medicine and environmental remediation has led to concerns regarding exposure of these nanoparticles to the public. However, limited studies are available to evaluate their effects on the environment, in particular on plants and food crops. Here, we investigated the effects of positive (PC) and negative (NC) charged iron oxide (Fe_2_O_3_) nanoparticles (IONPs) on the physiology and reproductive capacity of *Arabidopsis thaliana* at concentrations of 3 and 25 mg/L. The 3 mg/L treated plants did not show evident effects on seeding and root length. However, the 25 mg/L treatment resulted in reduced seedling (positive-20% and negative-3.6%) and root (positive-48% and negative-negligible) length. Interestingly, treatment with polyethylenimine (PEI; IONP-PC coating) also resulted in reduced root length (39%) but no change was observed with polyacrylic acid (PAA; IONP-NC coating) treatment alone. However, treatment with IONPs at 3 mg/L did lead to an almost 5% increase in aborted pollen, a 2%–6% reduction in pollen viability and up to an 11% reduction in seed yield depending on the number of treatments. Interestingly, the treated plants did not show any observable phenotypic changes in overall size or general plant structure, indicating that environmental nanoparticle contamination could go dangerously unnoticed.

## 1. Introduction

Nanotechnology is a fast developing industry that grew from $4 billion in investments in 2005 to more than $224 billion of manufactured goods in 2009 [1,2,3]. Expected production of engineered nanoparticles (NPs) is projected to reach 58,000 tons in 2011–2020 [4,5]. Engineered NPs are usually divided in four main categories: nanosized polymers, carbonaceous NP, metal-based NPs that include metal oxide and quantum dots, and composites integrating NPs [6]. NPs of 1–100 nm have attracted the most interest due their unique properties that are not present in corresponding bulk materials [7,8,9] including quantum confinement, a large surface area to volume ratio, high surface energy, and several other catalytic and magnetic properties [10,11]. Controlling the release of engineered NPs into the environment has proven difficult due to the rapid growth of the nanomaterial industry and the usage of nanomaterials in a wide array of products [12].

Plants are an essential component in ecological systems and may serve as a potential pathway for NP transport into the food chain and a route for bioaccumulation in higher organisms [13,14,15,16]. Many of the reported studies in the literature are focused on silver (Ag) and titanium oxide (TiO_2_) NPs because of their extensive use in food packing and cosmetics [13,14,15,16,17,18,19,20,21]. Most of the current published studies regarding NPs and plants are centered around the effects of NPs on seed germination and vegetative plant growth [6]. Depending on the types of NPs and the plant species under study, both positive and negative effects have been reported [22,23,24,25,26]. The effects observed are highly dependent on the type and concentration of NPs as well as the plant species and growth conditions used in the experiments. For example, carbon nanotubes were shown to be able to penetrate plant seed coats and dramatically affect both seed germination and plant growth [27]. TiO_2_ NPs were reported to improve the growth of spinach by enhancing their photosynthetic rate and nitrogen-fixation capacity in leaves and roots [14]. In contrast Zinc oxide NPs exhibited inhibitory effects on the germination and growth of plants. Ag NPs, the most prevalent metallic NPs in consumer products, showed no effects on the biomass and transpiration volume of zucchini plants [6]. However in another study, phytotoxicity of Ag NPs on plant seedling and growth at low concentration was observed and the phytotoxicity was concentration and size dependent [14]. A developmental phytotoxicity study [16] indicated that zinc oxide NPs were most phytotoxic, followed by magnetite, silica and alumina NPs, which were not toxic. Experiments by Lopez-Moreno *et al.* [28] showed that the germination rate and growth of soybean plants was enhanced after treatment with silicon oxide (SiO_2_) and TiO_2_ NPs. The authors hypothesized that the observed growth enhancement might be mediated by NP treatment promoting the ability of soybean to absorb water and by enhancement of overall nitrate reductase activity in the plants [28,29]. The same study also showed that application of cerium oxide NPs (CONPs) had genotoxic effects on soybean plants [28]. Additional work by the same group has shown that exposure to CONPs significantly reduced corn, tomato and cucumber germination [30]. CONPs also significantly inhibited root growth in both alfalfa and tomato but enhanced the root growth in cucumber and corn. At the same time CONPs significantly increased root and stem growth in corn, alfalfa, and soybean when applied at concentrations of 500, 1000, 2000, and 4000 mg/L [30]. Clearly there is no consensus regarding the effects of a wide variety of NPs on plant growth and development.

Upon uptake by plants, NPs can be transported and localize in various tissues. For instance, a significant amount of iron oxide NPs suspended in liquid media were shown to be taken up by pumpkin roots and translocated throughout the plant tissues [31]. However, the NPs were primarily accumulated near the root and only a small percentage was detected in the leaves, due to the large size range (30 nm–1 µm) of the commercial iron oxide NPs [31]. Zinc oxide NPs were reported to pass through the root epidermis and cortex via the apoplastic pathway through cell walls [32]. Carbon-coated iron NPs injected or sprayed at certain locations on pumpkin leaves were shown to be capable of transport to other locations in the plant [33]. Detailed microscopic analysis revealed the presence of NPs in pumpkin xylem vessels, a major water transporting tissue in plants [33]. Another report demonstrated efficient delivery of DNA and chemicals through silica NPs internalized into plant cells [34]. In contrast, a study on CONPs did not show any evident translocation of NPs in maize plants [23]. Even with inconsistent results and unclear mechanisms, it is certain that plants can take up NPs and that NPs can be localized inside plant tissues. However, what remains unclear is the ultimate fate and physiological/reproductive effects of these NPs.

Iron oxide nanoparticles (IONP) are widely used in biomedicine for drug delivery and in magnetic resonance imaging (MRI) [35,36,37,38,39]. These nanoparticles are also widely used for a variety of other applications, such as soil and groundwater treatments [40] and photocatalytic reactions [41,42]. Iron oxide nanoparticles are also generated from the oxidation of zero-valence iron NPs, which have been used for environmental remediations [43,44,45,46,47], with more than 50 commercial sites in the United States [48]. The increasing commercial use of IONPs has resulted in a concomitant accumulation of them in the environment. Iron oxides are also present in nature as nano-sized crystals as both maghemite (Fe_2_O_3_) and magnetite (Fe_3_O_4_) formed naturally by fire events and volcanism. A major question is how detrimental these higher concentrations of IONPs are to the environment—especially considering that many of these IONPs are coated with biocompatible molecules [49].

In this study we have investigated the effects of spherical, charged IONPs (Fe_2_O_3_) nanoparticles on growth, development and seed production in the model plant *Arabidopsis thaliana* L. Heynh. *A. thaliana* is an exceptional model for experimental work because it contains a small, diploid genome making it tractable for genetics and genomics, it is self-fertile and produces copious amounts of seeds (up to 10,000 per plant), large numbers can be cultivated in a small space and plants can be easily transformed using *Agrobacterium tumefaciens* [50]. Additionally, *Arabidopsis* has numerous genetic and genomic resources that are publically available and has been used in several other NP studies [51,52,53,54]. Here, we exposed plants to negatively charged IONPs (IONP-NC) coated with polyacrylic acid (PAA) groups and to positively charged IONPs (IONP-PC) that had a coating of polyethylenimine (PEI) groups. Previous studies have shown that the surface coatings of NPs significantly impact their cytotoxicity [55,56,57]. Both of the charged coatings used in the current study greatly increase water solubility of the NPs potentially expanding their effects in living organisms [58]. To the best of our knowledge, there are no data directly showing the effects of IONP with charged coatings on plants. In the current study we show that both IONP-NC and IONP-PC have significant effects on *A. thaliana* root and shoot growth, pollen viability and overall seed production.

## 2. Results and Discussion

### 2.1. Absorption and Translocation of Nanoparticles in A. thaliana

**Figure 1:**
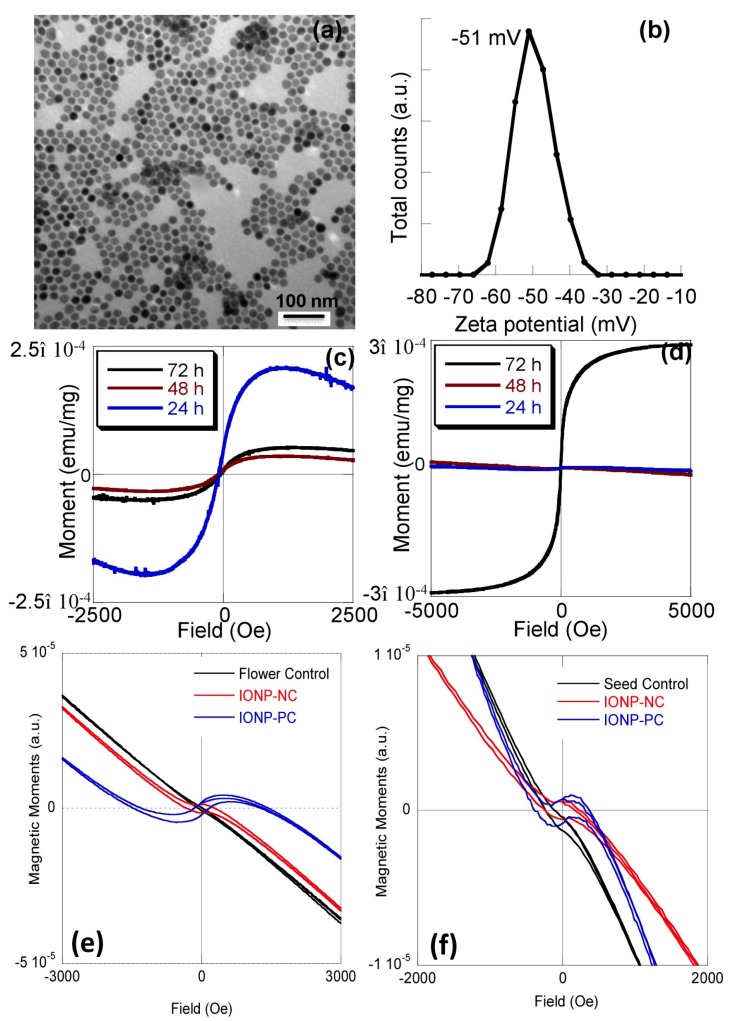
Transmission electron micrograph (TEM) imaging and magnetic moment measurements of iron oxide nanoparticles (IONP) transport in *A. thaliana* tissues. (**a**) TEM image of the iron oxide NPs used in this experiment, showing spherical shape; (**b**) Zeta potential plot of the IONP-NC solution; (**c**) Magnetic measurements of the root tissues at 24, 48 and 72 h after watering with 30 mL of a 3 mg/L IONP-NC; (**d**) magnetic measurement of the leaf tissues at 24, 48 and 72 h after watering with 30 mL of a 3 mg/L IONP-NC; (**e**,**f**) magnetic moment measurements of IONP-PC and IONP-NC in flowers (**e**) and seeds (**f**).

Iron oxide nanoparticles were prepared following our previously established method [58]. The nanoparticles have a core size of 12 nm and hydrodynamic size of 28 nm for PAA-coated and 30 nm for PEI-coated nanoparticles. Figure 1a shows a transmission electron microscopy image of typical IONP-NC with a narrow size distribution. The surface charge of the NPs can be effectively determined with the zeta potential plot in water (pH 7.0), as shown in Figure 1b. In addition to the surface charge, the value of −51 mV also indicated the stability of the NP water dispersion, because NP dispersion is generally considered stable when its zeta potential value is higher than +30 mV or lower than −30 mV. For nanoparticle treatment, twenty-one day old *A. thaliana* plants were watered with a solution of water (pH 7.0) containing negatively charged PAA-coated IONPs (IONP-NC) at a concentration of 3 mg/L (~1.0 mg/L total iron). The concentration for these initial studies was chosen because this is the physiological concentration of iron required as a micronutrient for plants. Plant tissues were harvested at different time intervals and in various tissues including roots and leaves (24, 48, and 72 h), flowers (4–5 weeks) and seeds (6–7 weeks) after watering with the IONP-NC solution. Roots were rinsed with distilled water to ensure that only IONPs taken up by the plant tissue would be measured. The tissue localization of NPs was studied by measuring the magnetic moments of the collected tissues using an alternating gradient force magnetometer. Only the presence of iron oxide NPs will show a magnetic signal, because the tissues themselves are diamagnetic. An initial high level of IONP-NC was present in the root tissue 24 h after watering and decreased significantly at the 48 and 72 h time points (Figure 1c). In contrast, the magnetic moment of IONP-NC in the leaf tissue at 72 h after watering was significantly higher than that of 24 and 48 h tissues (Figure 1d). In addition to the high level of IONP-NC accumulation in the leaves, we also observed localization of both IONP-PC and IONP-NC inside flowers and seeds (Figure 1e,f), indicated by the enhanced positive magnetic moments compared to the control flowers and seeds. For simplicity, we are only showing the root and leaf time course data for IONP-NC in Figure 1 (Figure 1a,b). Data obtained for IONP-PC in roots and leaves showed exactly the same trends as that for IONP-NC. Taken together, these data show that charged IONPs are efficiently transported into the plant from soil and are then mobilized from the root tissue into aerial portions of the plant.

The uptake and transport of IONPs has been previously shown in pumpkin by Zhu *et al.* [31] though their study utilized Fe_3_O_4_ IONPs. Additionally this study only detected the absorption of IONP if the plants were grown hydroponically. No NP absorption was reported when pumpkin plants were grown in soil or sand [31]. Several mechanisms for the absorption of NPs by plants have been proposed [59]. It has been hypothesized that because of their high surface area, NPs could bind to carrier proteins or to organic chemicals present in the media and be absorbed with them [59]. Also, it is widely thought that the absorption of most metal-based nanoparticles occurs with partial involvement of the numerous ion transporters present in the plant cell membrane [60]. However, an exact mechanism for the absorption of IONP has yet to be reported and more detailed studies on these processes are required. Clearly the individual plant species, the size and charge of the NPs and the growth media all influence the ability of IONPs to be transported into plants [61].

### 2.2. Effect of Iron Oxide Nanoparticles (IONPs) on Seed Germination, Seedling and Root Length

Since our data indicated that charged IONPs were transported into all major tissues of the plant, we hypothesized that IONPs might affect overall growth and development in plants. To determine this we examined both seed germination, seedling length and root elongation in *A. thaliana* grown on agar media in the presence of IONP-PC or IONP-NC. Overall seed germination rates along with seedling and root elongation are standard assays used to determine phytotoxicity for environmental biomonitoring [62,63]. Studies have demonstrated that the sensitivity of these assays, particularly for metal ion toxicity, is increased if the assays are performed on agar media [64].

*A. thaliana* Col-0 ecotype seeds were grown on agar plates containing 3 mg/L of IONP-PC or IONP-NC and compared with water controls. No differences were observed in seed germination rates, overall seedling length or root length in seedlings germinated and grown on media containing 3 mg/L of either charged IONP (data not shown). This observation was likely due to the low concentration of the charged IONPs in the media. Subsequently, the concentration of charged IONPs was increased to 25 mg/L (8 mg/L total iron) based on other published studies in the literature. No statistically significant difference was observed in the germination rate of seeds grown on media containing either IONP-PC or IONP-NC at 25 mg/L (data not shown). We attributed this observation to the seed coat, which has selective permeability and is an important and very effective barrier. Other studies have shown that mutations that increase seed coat permeability can have negative effects on seed germination rates [65]. Lin and Xing [32,66] showed that exposing plants to zinc oxide NPs resulted in no significant effect on seed germination in five out of six studied plant species. In our study, the IONPs did not cause a decline in seed germination most likely because they were unable to traverse the mature seed coat. However, seedlings were more vulnerable to IONPs after germination since the NPs were directly absorbed by the developing root system.

**Figure 2:**
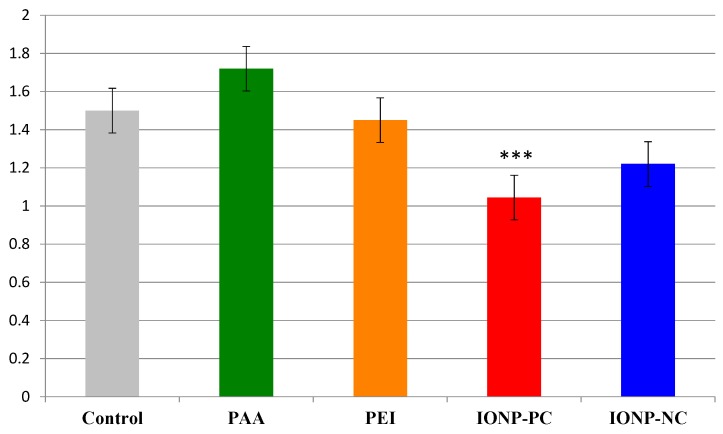
Effect of IONP treatment on overall seedling length in *A. thaliana*. Mean seedling length of plants grown on agar media containing either IONP-PC (red), IONP-NC (blue), PAA only (green) or PEI only (orange) compared with agar control (gray). *A. thaliana* Col-0 seeds were grown in MS media supplemented with IONPs at 25 mg/L and PEI or PAA at 12.5 mg/L. Seedling lengths were measured six days after planting. Treatments marked with asterisks were significantly different from the control with *p* < 0.0001. For all treatments *n* = 140.

In contrast to the germination data, *A. thaliana* seedlings that were germinated on plates containing 25 mg/L IONP-PC did have a statistically significant reduction in overall seedling length compared to seedlings that germinated in MS media only (Figure 2). IONP-PC reduced the length of *A. thaliana* seedlings by about 20% on average (*p* = 4.098 × 10^−8^). In contrast, IONP-NC reduced seedling length by only 3.6% (*p* = 0.28), which was not significantly different from the control treatment (Figure 2). Additionally, seedlings were treated with nanoparticle coatings alone as a negative control at a concentration of 12.5 mg/L which is equivalent to the amount of coating present on the nanoparticles at a concentration of 25 mg/L. Treatment of seedlings with either PAA or PEI coating alone did not have a statistically significant effect on seedling length when compared to agar only controls (Figure 2).

**Figure 3:**
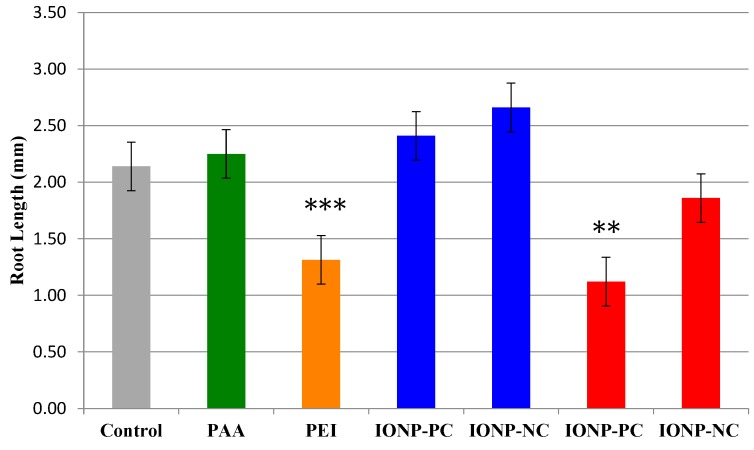
Changes in *A. thaliana* seedling root length after exposure to IONPs. Seeds were grown in MS media supplemented with either INOP-PC or IONP-NC at concentrations of 3 µg/mL (blue) or 25 µg/mL (red); or PAA only (green) or PEI only (orange) at 12.5 mg/L and compared with growth media only control (gray), Root lengths were measured six days after planting. Treatments with significant difference of *p* < 0.009 are marked with two asterisks; *p* < 0.0001 are marked with three asterisks. For all treatments *n* = 74.

A second series of experiments was performed to measure root length of seedlings germinated on media containing either 3 or 25 mg/L charged IONPs six days after planting. Only seedlings grown on plates with either 25 mg/L IONP-PC or 12.5 mg/L PEI showed a significant reduction in their root length (Figure 3) with a reduction of about 48% in the IONP-PC treatment (*p* = 0.003) and a 39% reduction on average with PEI treatment (*p* = 1.6 × 10^−6^) No other treatments resulted in a reduction in root length (Figure 3) reinforcing the results observed on seedling length in IONP treated plants though it appears that most of the reduction in root length is due to the PEI coating alone. Similar developmental changes in *A. thaliana* seedlings were also observed using silicon oxide nanoparticles in a study by Lee *et al.* [16]. Exposure to high concentrations of silicon oxide NPs (4000 and 2000 mg/L) was shown to significantly reduce root elongation in *A. thaliana*. However, a significant increase in the root length was recorded if plants were treated with lower concentrations (400 mg/L) of silicon oxide NPs [16]. The authors of this study also exposed *A. thaliana* to other types of NPs, including iron nanoparticles (Fe_3_O_4_). Fe_3_O_4_ nanoparticles at all three concentrations tested (400, 2000 and 4000 mg/L) resulted in significant inhibition of root length in *A. thaliana*. Possible rationales for the difference in our current results and those of Lee *et al.* [19] are the much higher concentrations of NPs used in their experiments and the difference in the atomic composition of the nanoparticles used in each study (Fe_3_O_4_ *vs.* Fe_2_O_3_).

Studies using NPs composed of Fe_3_O_4_, Fe_2_O_3_ (coated with citric acid and non-coated) and nano-zero valent iron (nZVI) have all shown that the phytotoxic effects of iron nanoparticles are highly dependent on the plant species and the atomic composition of iron nanoparticles [67,68,69]. Sarvendra-Kumar *et al.* [67] showed that at low concentrations of Fe_2_O_3_ nanoparticles (10 to 40 mg/L) there was a slight increase in the length of shoots and roots of rice, wheat, cucumber and mung bean. However, higher concentrations of the same nanoparticles were shown to inhibit the growth of both roots and shoots in all four plant species tested. In contrast, Alidoust and Isoda reported only positive growth effects of Fe_2_O_3_ nanoparticles on root length in rice and soybean at high concentrations (1000 and 2000 mg/L) [69,70]. The critical difference between these studies was the actual size of the nanoparticles used in the experiments. Sarvendra-Kumar *et al.* [67,69] used nanoparticles that had a particle size of 50 nm while Alidoust and Isoda used much smaller nanoparticles (6 nm). These results emphasize how critical the size of nanoparticles can be in the phytotoxic effects observed in plant species.

### 2.3. Effects of IONPs on Pollen Viability and Pollen Tube Growth

In addition to the study of NP effects on seedling and root length, the long-term effects of charged IONPs on plant reproductive structures were examined *in vivo*, including pollen viability and pollen tube length*.* Pollen viability is a well-established biomarker for the investigation of toxic effects of materials on plant reproduction [71].

To determine the detrimental effects of IONPs on pollen development, *A. thaliana* plants were dosed with 3 mg/L IONP-NC, IONP-PC or distilled water 14 days after planting (dap) or 14 and 21 dap. Pollen was harvested from flowers of treated plants (floral stage 12) and subjected to Alexander’s staining to test pollen viability. The dH_2_O control, IONP-NC and IONP-PC treated plants all grew to approximately the same height and produced similar numbers of flowers and siliques. The number and formation of the floral structures was not impacted by charged IONP treatment (data not shown). Pollen from plants treated once with both IONP-NC and IONP-PC showed a slight increase in the ratio of aborted pollen compared to controls (Table 1; *p* < 0.0001). The largest increases in aborted pollen occurred in plants that were treated with charged IONPs twice, on days 14 and 21. The ratio of aborted pollen increased 4.2% in IONP-PC treated plants and 4.0% in plants that were treated with IONP-NC (Table 1; *p* < 0.0001). All plants that were treated with IONPs showed a statistically significant increase in aborted pollen compared to the dH_2_O control plants no matter how often the plants were treated with NPs or the charge on the NPs (Table 1). Pollen viability testing of IONP treated plants using aniline blue staining showed that only plants treated with IONP-NC at 14 and 21 dap have a reduction in viability of 4% compared to control pollen (Table 2; *p* < 0.05).

**Table 1:** Treatment of *A. thaliana* plants with IONPs results in increased amounts of aborted pollen.

Treatment Day	dH_2_O Control	IONP-PC	IONP-NC
14	2.98%	4.07% ***	4.26% ***
14 & 21	2.73%	6.94% ***	6.77% ***

*A. thaliana* Col-0 plants were treated with charged IONPs at a concentration of 3 mg/L or dH_2_O on indicated days after planting (dap). Pollen from newly opened flowers was harvested and subjected to Alexander’s staining per published protocols [72]. Numbers represent the percentage of aborted pollen observed in each treatment. Seven flowers from nine independent plants were observed (~7000 pollen grains) with two independent replications for each treatment. Treatments marked with three asterisks were significantly different from the control with *p* < 0.0001.

**Table 2:** Percentage of viable pollen in IONP treated *A. thaliana* using aniline blue staining.

Treatment Day	dH_2_O Control	IONP-PC	IONP-NC
14	98.8%	98.1%	98.3%
14 & 21	97.6%	93.3% *	98.1%

Aniline blue staining of *A. thaliana* treated with charged IONPs at a concentration of 3 mg/L or dH_2_O on indicated days after planting (dap). Pollen was harvested form 4 week old plants, stained with aniline blue and observed under a microscope. Numbers represent the percentage of viable pollen observed in each treatment. Seven flowers from nine independent plants were observed for each treatment (~7000 pollen grains) with two independent replications for each treatment. Single asterisks represent treatments that were significantly different from the control with *p* < 0.05.

**Figure 4:**
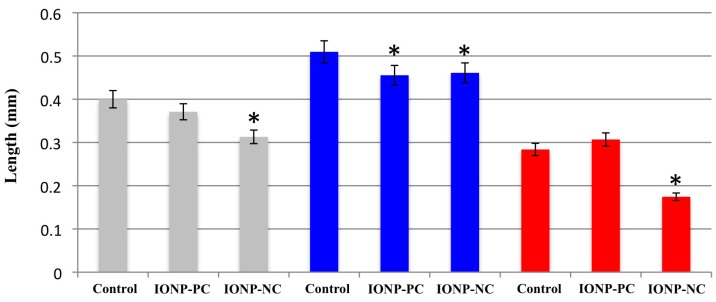
Effects of IONPs on Pollen tube growth in *A. thaliana*. Plants were treated with 30 mL of a solution containing IONP-PC or IONP-NC at a concentration of 3 mg/L either once (14 dap; gray bars); twice (14 & 21 dap; blue bars) or three times (14, 21 & 28 dap; red bars) and compared with dH_2_O only controls. Pollen was then collected from fresh flowers of treated plants and grown on pollen tube germination media for 18 h before pollen tube length was measured. Treatments with significant differences from the control are marked with an asterisk (*p* < 0.05). For all experiments *n* = 100.

Our studies clearly showed that pollen viability was affected by the treatment of charged IONPs. Subsequently we tested whether pollen germination and pollen tube length were also altered by IONP treatment. Successful fertilization between the ovule and sperm in plants requires both viable pollen and the successful formation of a pollen tube to deliver the sperm cell to the ovule. If pollen tube formation is affected by IONPs then this could lead to a loss of successful fertilization events translating into lower seed yields. For these experiments, pollen was harvested from plants treated with 3 mg/L of either IONP-PC or IONP-NC 14 dap, 14 and 21 dap or 14, 21 and 28 dap. Pollen from treated plants was harvested from newly opened flowers and grown on pollen tube germination media for eighteen hours. Individual pollen tube length was then measured using light microscopy and imaging software. Pollen from plants treated one time with IONP-NC 14 dap was 25% shorter than that of the dH_2_O control group (Figure 4). While less dramatic, pollen tubes of plants treated two times (14 and 21 dap) with either IONP-PC or IONP-NC were also significantly shorter than that of the water control group (Figure 4). Pollen tubes from plants treated three times with IONP-NC (14, 21, and 28 dap) were over 50% shorter than that of the control group (Figure 4). Along with the observed reduction in pollen viability, these results suggest that charged IONPs negatively impact plant reproductive capability.

Our experiments *in vivo* demonstrated that both IONP-NC and IONP-PC have negative effects on pollen viability and pollen tube growth in *A. thaliana* even at low concentrations of nanoparticles (3 mg/L)*.* The observed phytotoxic response was dependent on the number of times plants were treated with nanoparticles and on the charge of the IONP. Plants that were treated one time with NPs (14 dap) showed a significant decrease in both pollen viability and pollen tube length when treated with IONP-NC (Table 1 and Figure 4). The most significant effects on pollen viability occurred when plants were treated twice with charged IONPs (Table 1). Statistically significant reductions in pollen viability, pollen tube length, and seed production were observed in the 14 and 21 dap treatment with both types of charged IONP. Our data showed that the amount of aborted pollen more than doubled in comparison with the control treatment. In addition, aniline blue staining of pollen also showed a significant reduction in pollen viability when plants were treated twice (14 & 21 dap) with IONP-PC (data not shown).

We observed significant differences in the amounts of aborted pollen observed depending on the staining method used (Alexander’s *vs.* Aniline Blue). In our experiments, the results obtained with Alexander’s staining (Table 1) showed significant reductions in pollen viability in a larger number of IONP treatments than did the aniline blue results. Our results with Alexander’s staining also correlate well with our pollen tube length data. Alexander’s stain contains acid fuchsin that stains the cytoplasm and malachite green that stains cellulose present in the pollen cell wall [72,73,74]. Pollen that has been aborted does not have a developed cytoplasm so only the cellulose will be stained blue. In live pollen the cytoplasm is stained red and the cellulose in the pollen wall is blue. Therefore Alexander’s staining allows for the detection of pollen that is aborted at an early stage of development [75]. This distinction is not possible with aniline blue since the lactophenol stains only the callose in the pollen wall [76]. In *A. thaliana* pollen development, callose is required the formation of the outer wall of the pollen but it is not required for pollen tube growth [77]. We hypothesize that this difference in the staining methods explains the fact that our observed results for pollen viability using Alexander’s staining are more closely correlated with the pollen tube germination results (Table 1 and Figure 4). Further this data appears to indicate that both types of charged IONP inhibit pollen cytoplasm formation but only IONP-NC had a significant effect on the pollen wall formation. These findings may be due to the different localization capacities of IONP in *A. thaliana* cells based on charge difference. A difference in cerium oxide and iron oxide NP localization in mammalian cell lines was observed by Asati *et al.* [78] based on NP charge. Interestingly, even though a difference in cellular localization was observed the study did not reveal any cytotoxic effects of IONP in cardiac myocytes or lung carcinoma cell lines [78].

### 2.4. Exposure to IONP Results in Changes in Overall Seed Yield in A. thaliana

**Figure 5:**
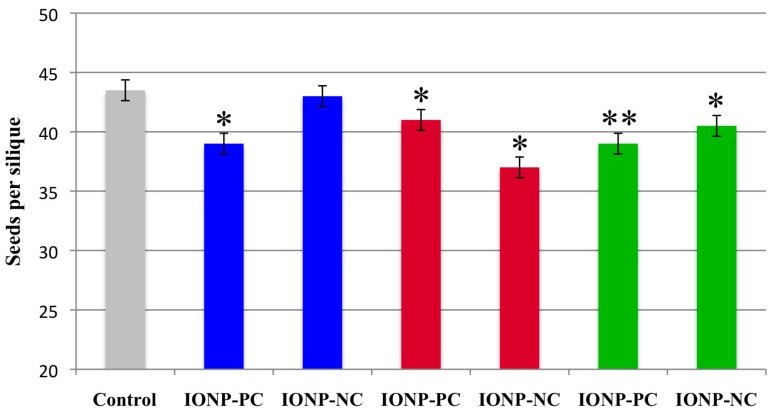
Treatment of *A. thaliana* with IONPs results in reduced seed production. *A. thaliana* Col-0 plants were grown in soil and watered once (14 dap, blue) twice (14 & 21 dap, red) or three times (14, 21 & 28 dap, green) with 30 mL of IONP-PC or IONP-NC at 3 mg/L and compared with dH_2_O only controls (gray). For all experiments *n* = 1200 siliques per treatment. Single asterisks represent treatments that were significantly different from the control with a *p* < 0.05; two asterisks indicate treatments that were significantly different with a *p* < 0.01.

Since pollen development is directly related to plant fertility, we further studied the effects of IONPs on the overall fertility in plants. To the best of our knowledge, no previous studies were reported on the effect of any metal oxide nanoparticles on plant fertility. Fertility and overall seed production is an important biomarker because it directly correlates not only with reproductive success in plants but also with the production capability of economically important crops. We directly examined fertility in *A. thaliana* by counting the average number of seeds each silique (seed pod) of an individual plant produced. *A. thaliana* plants typically produce 20–30 siliques per floral stem with each silique containing about 40–45 seeds. Most healthy plants produce 5–6 floral stems. For these experiments, plants were treated with IONP-PC or IONP-NC at a concentration of 3 mg/L. Plants were dosed with the charged IONPs either once (14 dap), twice (14 and 21 dap) or three times (14, 21 and 28 dap) and allowed to grow to maturity (5 weeks after planting). Siliques were then harvested, dried and numbers of seeds produced per silique were recorded. Plants from both charged IONP treatments exhibited a significant reduction in seed production except plants that were treated with IONP-NC 14 dap (Figure 5). In plants treated two times with charged IONP (14 and 21 dap), exposure to IONP-NC resulted in a more significant reduction in seed numbers than plants treated with IONP-PC (Figure 5). Interestingly, in plants dosed three times with charged IONPs (14, 21 and 28 dap) seed production was most reduced (11% reduction; *p* < 0.01) in the IONP-PC treatment, the reverse of what was observed in the 14 and 21 dap treatment. The exact reason for the difference in toxicity between the treatments with charged IONP is not clear at this time though we hypothesize that differences in the ability of the charged molecules to cross cell membranes, the impermeability of the developing seed coat and the buildup of the charged IONPs in tissues at the critical stages of development may play a role. It is doubtful that ion release from the IONPs is responsible for the observed toxicity differences since the pH of the *A. thaliana* cytosol is 7.3 and this is not low enough to cause ion release from the IONP coatings [79]. Nevertheless, these data clearly indicate that exposure of *A. thaliana* to low concentrations of charged IONPs has a detrimental effect on overall seed yield.

## 3. Experimental Section

### 3.1. Iron Oxide Nanoparticle Synthesis and Ligand Exchange

Iron oxide nanoparticles (NP) were synthesized and processed using methods previously described [58]. Briefly, NP synthesis occurred via heating the iron oleate complex in 10 mL 1-octadecene in presence of trioctylphosphine oxide/oleic acid (TOPO/OA) (TOPO: 0.2 g, 0.5 mmol; OA: 0.22 mL, 0.7 mmol). The reaction mixture was kept at 100 °C for 1 h to remove residual solvents prior to heating to 320 °C. The reaction mixture was reacted at 320 °C for 2.5 h before it was cooled down to room temperature. The NPs were then centrifuged down and dried under vacuum overnight. The desiccated powder was dispersed in chloroform under sonication to produce 5 mg/mL stock solution. The ligand exchange process was achieved by mixing the desirable ligands with NP stock solution in dimethyl sulfoxide (DMSO) at room temperature for 48 h. 1 mL stock solution was mixed with polyacrylic acid (PAA, 5 kDa), or polyethylenimine (PEI, 10 kDa) in 50 mL DMSO. The reaction proceeded for 48 h and then the NPs were magnetically collected and diluted in 5 mL water, producing a NP solution with a concentration of 1 mg/mL. Here, the NP weight includes the surface coating. Two types of spherical, charged Fe_2_O_3_ nanoparticles were used in this investigation: negatively charged PAA coated NPs and positively charged PEI-coated NPs.

### 3.2. Biological Materials

*Arabidopsis thaliana* L. Heynh ecotype Columbia (Col-0) was used for all experiments in this study. Seeds were obtained from laboratory stock, which originated from *A. thaliana* Col-0 seeds purchased from Lehle Seeds (Round Rock, TX, USA). All seeds were stratified at 4 °C to synchronize seed germination for experiments.

### 3.3. Seedling and Root Length Experiments

Seeds were surface sterilized by agitating in a solution of 50% commercial Clorox bleach (8.25% sodium hypochlorite in water) for 5 min, followed by a 70% ethanol wash for 2 min and then rinsed 3 times with distilled water. All germination experiments were performed in 100 × 15 mm petri dishes containing Murashige and Skoog basal medium supplemented with Gamborg’s vitamins at pH 5.7. For the IONP-PC, IONP-NC, PEI and PAA treatments, warm MS media (0.8% agar) was poured into petri dishes and solutions containing the IONPs at concentrations of 3 or 25 mg/L or PAA and PEI coatings at 12.5 mg/L were immediately added to the warm media and swirled to ensure even distribution across the plate. Each plate contained approximately 40 mL of media and 40 Col-0 seeds with a minimum of 1 cm between them were sown on the plate surface. Plates were then placed in a Percival growth chamber (24 °C day, 22 °C night, 16 h day, 150 µE·m^−2^·s^−1^ of light, 60% relative humidity) for 6 days at 24 °C. After 6 days, total seedling length (from the tip of the root to the terminal bud) or root length was measured using a stereomicroscope.

### 3.4. Nanoparticle Treatment of Plants Grown in Soil

*A. thaliana* Col-0 seeds were surface sterilized as previously described and planted in sterile PRO-MIX HP (Premier Horticulture, Quakertown, PA, USA) growth medium for all pollen viability, pollen tube germination and seed production studies. PRO-MIX HP is composed of Sphagnum peat moss (73% by volume), Horticultural grade perlite (27% by volume), calcitic limestone, dolomitic limestone, and a wetting agent. For these experiments each plant was separated into individual pots so that each plant was isolated from the other plants in the experiment. Flats of pots were placed into a Percival growth chamber for 7 days to allow seedlings to germinate (24 °C day, 22 °C night, 16 h day, 150 µE·m^−2^·s^−1^ of light, 60% relative humidity). After 7 days, plants were transferred to light banks (24 °C day, 22 °C night, 16 h day, 300 µE·m^−2^·s^−1^ of light) and grown to maturity. Each plant was watered with 30 mL of 3 mg/L solution of IONP-PC or IONP-NC suspended in distilled water at pH 7.0 or with dH_2_O pH 7.0 for the controls. Plants were treated with the solutions either one time at 14 dap, two times at 14 and 21 dap, or three times 14, 21, and 28 dap.

### 3.5. Alexander’s Stain and Aniline Blue Staining for Pollen Viability

A simplified version of Alexander’s staining proposed by Peterson *et al.* [80] was used to test pollen viability. A key advantage of this method is the exclusion of highly toxic phenol from the stain. At 35 dap, all flower buds at stage 12 [81] were collected and preserved with Carnoy’s fixative (6:3:1 alcohol:chloroform:acetic acid) [82]. Buds were preserved for almost two months in fixative. Floral buds were dissected under a stereomicroscope and the anthers were removed before staining. Anthers were then carefully crushed to release the maximum amount of pollen. Pollen was stained using protocols described by Peterson *et al.* [80]. After staining, all aborted and non-aborted pollen was counted using a Zeiss compound microscope at 200× magnification.

Pollen viability was also tested using aniline blue staining as previously described [74,76,83]. Flowers at stage 12 were harvested 35 dap. Newly opened flowers were individually touched to glass slides containing 20 µL aniline blue-lactophenol stain [83]. The stain was then diluted 1:1 with distilled water. Pollen grains were examined under a stereomicroscope at 10× magnification. Dark or navy blue grains were considered viable, clear or light blue grains were counted as non-viable.

### 3.6. Pollen Tube Germination

Pollen tube germination experiments were performed as described previously [84]. In brief, pollen from freshly opened stage 12 flowers was harvested from distilled H_2_O control and IONP treated plants 35 dap. Pollen from flowers was directly placed onto pollen tube germination (PTG) media by touching the anthers to the media in petri dishes. PTG media contains 18% *w*/*v* sucrose, 0.01% *w*/*v* boric acid, 1 mM CaCl_2_, 1 mM Ca(NO_3_)_2_, 1 mM MgSO_4_, and 0.5% *w*/*v* agarose [84]. PTG media pH was adjusted to pH 7.0 (+/−0.1) before adding agar. Plates containing pollen were then incubated at room temperature for 18 h and the NIH ImageJ software package (Bethesda, MD, USA) was used to measure pollen tube length.

### 3.7. Statistical Analysis

All experimental results are presented as mean values with standard deviations shown. In all cases experimental treatments were compared to controls using a Student’s *t*-test with three independent biological replicates used for each experiment. Sample sizes for experiments are given in the figure legends. The statistical significance of results was determined using a minimum 95% confidence interval (*p* < 0.05) unless noted in the text or figure legends.

### 3.8. Seed Production Assay

Mature siliques were harvested simultaneously from *A. thaliana* plants (control and treated) 9 weeks after planting. Individual siliques were placed in Eppendorf tubes; the tubes were grouped by plant in freezer boxes and then allowed to dry. Siliques were then carefully opened and the seeds were extracted and counted for each plant. 1200 siliques were harvested from each treatment (IONP-PC or IONP-NC) and control (dH_2_O) plants. Seeds per silique were then counted for each experimental or control treatment.

## 4. Conclusions

In the current study we showed that IONP-PC and IONP-NC have inhibitory effects on development and reproduction in *A. thaliana*. Our data indicate that charged IONP are transported throughout the plant and can be detected in root, leaf, floral and silique tissue. IONP-PC had significant effects on seedling and root length in *A. thaliana* but most of the effect on root length could be attributed to the PEI coating on the nanoparticle. Significant effects of both positively and negatively charged nanoparticles were observed on pollen viability, pollen tube growth and seed production. These results clearly demonstrate that IONPs have a detrimental effect on overall plant reproduction. Additionally our results indicate that IONP toxicity is highly dependent on NP concentration and on the number of NP applications to plants. Further studies are needed to more precisely determine the stage of pollen and ovule development that is effected by IONPs. In addition, experiments to determine the molecular mechanisms underlying the absorption and toxicity of IONP in *A. thaliana* will be important. Further experiments based on this work will focus on economically important crop species (tomato, soybean) to determine the effects of IONPs in the environment on their yield.
